# Can breakfast tryptophan and vitamin B6 intake and morning exposure to sunlight promote morning-typology in young children aged 2 to 6 years?

**DOI:** 10.1186/1880-6805-31-11

**Published:** 2012-05-22

**Authors:** Miyo Nakade, Osami Akimitsu, Kai Wada, Milada Krejci, Teruki Noji, Nozomi Taniwaki, Hitomi Takeuchi, Tetsuo Harada

**Affiliations:** 1 Department of Nutritional Management, Faculty of Health and Nutrition, Tokai Gakuen University, Nagoya, Japan; 2 Laboratory of Environmental Physiology, Graduate School of Integrated Arts and Sciences, Kochi University, Kochi, Japan; 3 Department of Health Education, Faculty of Education, University of South Bohemia, Ceske Budejovice, Czech Republic; 4 Department of Physical Education, Faculty of Education, Kochi University, Kochi, Japan; 5 Affiliated Kindergarten, Faculty of Education, Kochi University, Kochi, Japan

**Keywords:** Morningness-Eveningness, Young children aged 2 to 6 years, Tryptophan and vitamin B6, Breakfast

## Abstract

This study tried to examine, from epidemiological and physiologic anthropological (Japanese culture on breakfast) points of view, the integrated effects of the amount of tryptophan and vitamin B6 intake and the following exposure to sunlight on the circadian typology and sleep habits in young Japanese children aged 2 to 6 years, using the newly-evaluated calculating system of tryptophan (Tryptophan Index 2009) and vitamin B6 intake (VitaminB6 Index 2009) at breakfast. The positive and significant correlation was shown between the Morningness-Eveningness (M-E) score and the Tryptophan Index and also the Vitamin B6 Index. This positive correlation between M-E score and amount of tryptophan intake was shown only by children who were exposed to sunlight for longer than 10min after breakfast. These results might support the following hypothesis: higher tryptophan and vitamin B6 intake at breakfast could promote the synthesis of serotonin via light stimulation in the morning in children.

## Introduction

Previous research has shown that diurnal rhythms and sleep health in early childhood may be highly sensitive to the living environment including light and regularity and timing of meals [1]. Tryptophan (Trp) is one of the essential amino acids absorbed exclusively from meals. It is metabolized to melatonin via 5-hydroxytryptamine (serotonin) by a series of four enzymes in the pineal body in which serotonin synthesis can be supported by vitamin B6 (Vi-B6) as a coenzyme in the tryptophan-serotonin metabolic pathway [2,3]. Hvans *et al.*[4] showed that a low plasma level of the metabolite of vitamin B6: pyridoxal 5’-phosphate (PLP, a coenzyme in the tryptophan-serotonin pathway) was significantly associated with the depression score. Volker *et al.*[5] showed in their review paper that Trp and vitamin B6 may serve as adjuncts to psychosocial and pharmacological therapies, with positive implications for long-term prognosis. Trp intake at breakfast has been shown to promote morning-typed circadian typology and higher sleep quality in Japanese children aged 0 to 6years [6], and such effect was enhanced by sunlight exposure after having breakfast [7]. The exposure to sunlight in the morning can be hypothesized to accelerate the synthesis of serotonin from Trp in the pineal gland [8].

Our research group previously calculated hypothetical trp intake from breakfast and analyzed how those values were related to M-E score and sleep habits. The results of the questionnaire survey showed that Trp intake at breakfast could make the sleep-wake habits of children aged 0 to 8 years (until 2nd year of elementary school) more morning-typed [6]. However, the estimation of Trp intake which was made and expressed as a Trp Index needed to be done more precisely, as the previous index did not include the amount of food per meal depending on age and it also should incorporate actual dishes currently eaten at breakfast by Japanese children.

In order to estimate more precisely how strong the correlation is between the amount of Trp and/or Vi-B6 consumed at breakfast and sleep habits and circadian typology in young Japanese children, the Trp Index (to estimate Trp intake) was modified and a new index to estimate Vi-B6 intake (Vi-B6 index) was created (Nakade *et al.*, unpublished). These two new indices incorporated the amount of food per meal depending on age and also actual dishes currently eaten at breakfast by Japanese children.

Diversification of lifestyles and advanced eveningness has created problems in the basic living habits of children during their growth period. This may contribute to a variety of adverse effects, such as a decrease in motivation for learning, a decrease in physical and mental energy, and possibly even an increase in several health disorders. Shifting to an evening-type life for young children might be being accelerated, especially due to the ongoing 24-h commercialization of society [9].

From the point of view of physiological anthropology, the Japanese consumed a Japanese-style breakfast which is protein (raw egg, dried fish, fermented soybeans, tofu in miso soup and so on) and vitamin-rich (dried seaweed, boiled spinach and so on). On the other hand, a light breakfast consisting of cereals, milk and vegetables are dominant and, for example, breakfast skippers are prevalent among female adolescents (23% skipped breakfast in 1999; 31.5% of all adolescents skipped breakfast in 2000 to 2006) and children frequently consumed ready-to-eat (RTE) cereal in the USA (35.9% of children aged 9 to 13years and 25.4% of adolescents aged 14 to 18years in 2000 to 2006) [10-12]. The number of students who had cereal and fruit juice for breakfast was increasing in Germany [13]. Protein consumption at breakfast by female Czech university students was significantly lower than female Japanese students (Kobayashi *et al.*, unpublished). Due to the ongoing 24-hcommercialization of society in Japan, the nutritional quality of breakfast can be predicted to worsen even in Japan. The results in this study can be passed on to promote physical and mental health in Japanese children.

This study aims to test the integrated effects of the amount of Trp and Vi-B6 intake and the following exposure to sunlight on the circadian typology and sleep habits in young Japanese children aged 2 to 6years, using the newly evaluated calculating system of Trp and Vi-B6 intake at the breakfast.

## Participants and methods

The data used were collected from responses to questionnaires completed in 2008 by children in nine city-run nursery schools and one kindergarten affiliated to the Faculty of Education, Kochi University located in Kochi city (33.3°N). The questionnaires included the Morningness-Eveningness Questionnaire (MEQ) by Torsvall and Åkerstedt [14] and a revised version for children [6], questions on the regularity of timing and contents of breakfast and nutritional balance, such as the frequency of having a meal that consisted of a staple food (carbohydrate), a main dish (protein), and a side dish (vitamins and minerals), questions on mental health of the children (such as anger and depression [6]) and questions about exposure to sunlight (the amount of time spent outside between the hours of awaking and arriving at school). The data from 816 children aged 2 to 5years (419 girls and 397 boys) were statistically analyzed using SPSS 12.0 statistical software.

### A. Questionnaire contents

#### Morningness-Eveningness Questionnaire

The MEQ [14] and a version for children [6] were used to objectively measure diurnal preference. This part consisted of seven questions: three pertaining to sleep onset, three to sleep offset, and once to the peak timing of activity. Each question allows for choice (scored from 1 to 4) and the M-E score was the sum of the seven answers. Scores ranged from 7 to 28, with lower scores representing evening-types and higher scores representing morning-types.

#### Sleep habits

This section consisted of questions on sleep onset and offset timings during weekdays and weekends and questions about the quality of sleep such as mood when falling asleep and waking up. In addition, participants were also asked about the types of bedroom curtains used to determine the sleep environment. The possible answers were: (1) no curtains and sunlight is able to freely enter the room through the window; (2) lace curtains; (3) regular (cloth) curtains; (4) blackout curtains (curtains with a high light-blocking effect that blocks almost all sunlight from entering the room when fully closed); (5) paper screens (Japanese *shoji*); and (6) blinds. With regards to curtains, participants were divided into two groups: for the sake of simplicity, participants using blackout curtains and blinds were put into a ‘blackout curtain group’ for those who use bedroom curtains with a high light-blocking effect (less than 10lx even in the daytime just behind the curtain), while all others were put in a ‘non-blackout curtain group’(more than 100lx in the daytime just behind the curtain).

#### Morning exposure to sunlight

1.How long is your child usually exposed to sunlight outside from the time he (she) has breakfast (or gets up) until he (she) arrives at nursery school (or kindergarten) (on days when they go to school; including time spent in the shade and clouded sunlight)? (1) Less than 10 min; (2) 10 to 30 min; (3) 30 to 60 min; (4) more than 60 min

2.How long is your child usually exposed to outside sunlight in the morning on days when he (she) has no school (including time spent in the shade and clouded sunlight)?

(1) Less than 10 min; (2) 10 to 30 min; (3) 30 to 60 min; (4)1 to 2 h; (5) 2 to 3 h; (6) more than 3 h

#### Breakfast habits

The questions were about the regularity of time when breakfast was taken, frequency of having a breakfast that consists of three components: a staple food (carbohydrate), a main dish (protein), and a side dish (vitamins and minerals); and the types of foods regularly eaten for breakfast.

#### Method for calculation of the estimated tryptophan and vitamin B6 intake

The calculation was done only for children who had breakfast every day or nearly every day. Children who ate irregularly and often skipped breakfast were deleted from the list for the calculation.

1. Amount of Trp or Vi-B6 contained in 100 g of food was estimated based on the tables on the contents of amino acids and vitamins in all kinds of foods [15]. On the other hand, the amount of food eaten by infants was estimated from the recommended amount of breakfast foods for children aged 2 to 5 years and general breakfast menu most frequently taken by Japanese young children [16,17]. The amount of Trp or Vi-B6 taken at one breakfast was calculated from the above two estimations (a-value).

2. The a-value was corrected based on the frequency (per week) of having a breakfast that consists of a staple food, a main dish and a side dish. The corrected a-value was defined as the revised index of Trp and Vi-B6. The following is how to calculate the revised index in detail.

#### Detailed explanation of how to calculate the indices based on amino-acid constituent tables

Based on the dietary intake standards for different age categories of Japanese children, recommended amounts were set for three levels: 1 to 2-year-olds, 3 to 5-year-olds, and 6 to 8-year-olds [16]. Children aged 1 and 6 years were excluded from this study; 1-year-olds are often in the post-weaning period and do not have established eating habits, and the number of 6-year-old respondents was too small.

A representative ‘menu’ for a well-balanced breakfast including a staple food, main dish, side dish and other (soup, beverages) was used for calculating the (Trp) and Vi-B6 indices. The Trp and Vi-B6 indices were adjusted based on the frequency of having a well-balanced breakfast. This adjusted value was calculated by reducing the value from 100% when a well-balanced breakfast was eaten every day, to 75% when it was eaten four to five times per week, to 50% when eaten two to three times, and to 25% when taken zero to onetime.

The validation test was done and the new indices for Trp and Vi-B6 (Spearman’s correlation test: Trp, r = 0.314 , *P* < 0.001, Vi-B6, r = 0.302, *P* < 0.001) showed a clearer difference in M-E scores than the former indices (Spearman’s correlation test: r = 0.256, *P* < 0.001, Vi-B6, r = 0.243, *P* < 0.001) on the same database.

#### Statistical analysis

Data were statistically analyzed using Spearman’s correlation test, the Wilcoxon signed-rank test, the Mann-Whitney U-test, and the Kruskal-Wallis test with SPSS 12.0 statistical software. M-E scores were expressed as mean plus or minus the standard deviation (Mean ± SD).

### B. Ethical treatment

The study followed the guidelines established by the journal *Chronobiology International* for the conduct of research on human subjects [18]. Before administering the questionnaires, each of the participants (parents or guardians) was given a written explanation that detailed the concepts and purposes of the study and stated that their answers would be used only for academic purposes. After the above explanation, all parents (or guardians) agreed completely with the proposal. The study was also permitted by the kindergarten nurses’ committees of the 13 kindergartens which carried out an ethical inspection of the contents of the questionnaire. As the children could not complete the questionnaires themselves, their parents or guardians completed them on their behalf.

## Results

**Figure 1:**
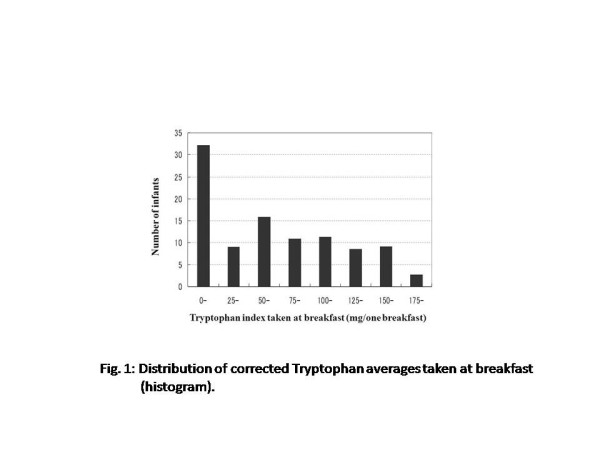
Distribution of corrected tryptophan averages taken at breakfast (histogram).

More than 90% of children have breakfast more than 5 to 6days per week, while 30% of those had a well-balanced breakfast including carbohydrate, protein, and vitamins and minerals with a frequency of once or never per week (Table 1). Figure 1 shows the distribution of the Trp averages taken at breakfast. When we incorporated the frequency information of taking the well-balanced breakfast per week to the Trp intake analysis, 32.0% of respondents had corrected Trp averages equaling 25.0 mg or less. There is a peak in the category of 50.0 to 75.0 mg with great variation in the values. This implies that there is a high amount of variation in the quality of breakfast among households.

**Table 1:** Breakfast habits of all participants

Regularity of breakfast								
	Eats at set times every day		Almost every day		Sometimes		Does not eat at set times	
*n*(%)	410 (50.6)		364 (44.9)		25 (3.1)		11 (1.4)	
Consumption of breakfast that includes staple, main and side								
	Everyday		4 to 5days/week		2 to 3days/week		0 to 1days/week	
*n*(%)	203 (25.1)		172 (21.3)		191 (23.6)		242 (30.0)	

Question: How many times a week does your child have a breakfast that consists of a staple food (e.g. rice, bread, noodles or potatoes), a main dish (e.g. meat, fish or eggs) and a side dish (e.g. vegetables)?

Table 2 shows the average Trp intake values according to the regularity of meal timings. Children who ate breakfast at the same time every day tended to have a higher Trp intake. Similarly, children who more frequently eat a well-balanced breakfast that contains a staple food, a main dish, and side dish tended to have higher Trp intake values (Table 2). These results agree with the assumption that families with established meal routines also have higher quality breakfasts. Even after running comparisons with other Trp indices, the corrected Trp averages in the revised version, Trp Index 2009, show the largest differences between the two groups, showing that these averages are currently the most appropriate index.

**Table 2:** New Trp Index (mg) based on breakfast habits and validation

**Corrected average Trp intake according to regularity of breakfast**						
	Eats at set times every day			Almost every day		
Aged 2 years		73.9	59.2			
Aged 3 to 5 years		83.8	62.4			
Corrected average Trp intake according to the frequency to take well-balanced breakfast that includes staple, main and side						
	Everyday		4 to 5 days/week	2 to 3 days/week		0 to 1 days/week
Aged 2years	134.4		98.0	46.8		12.0
Aged 3 to 5years	139.1		93.4	50.2		13.5
Trp-Index for children aged 3 to 5 years(Trp intake )before and after the correction due to the frequency of a well-balanced breakfast						
			Average after correction		Average before correction	
Eats at set times every day			83.8		125.5	
Almost every day			62.4		116.5	
Mann-Whitney U-test			z=-4.9,*P* <0.001		z=-2.7,*P=*0.006	

### Basic analysis

**Figure 2:**
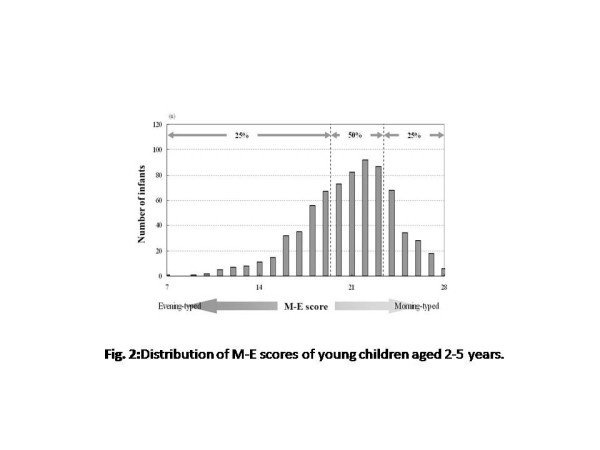
Distribution of M-E scores of young children aged 2 to 5 years.

The mean Morningness-Eveningness (M-E) score for all children was 20.80 ± 3.45 points (Figure 2). Dividing the children into age groups, the mean for 2-year-olds (173) was 22.1 ± 2.88 and for 3 to 5-year-olds (555) was 20.8 ± 3.51.

Table 3 shows the distribution of sleep habits on weekdays and weekends/holidays divided into age groups. Both bed times and wake-up times were later on weekends and holidays than on weekdays and both of these trends were greater for 3-5-year-olds than for 2-year-olds. No significant differences were seen for sleep duration between weekends/holidays and weekdays or between the different age groups. Regardless of age, 30.0% of children used a blackout curtain in their bedroom.

**Table 3:** Wake-up time, bed time and sleep hours divided by age groups (Mean ± SD[n])

	Aged 2 years	Aged 3 to 5 years	χ^2^value	df	*P*
Bed time					
Weekdays	21.21 ± 0.68(187)	21.45 ± 0.73(602)	39.61	31	0.138
Holidays	21.43 ± 0.69(181)	21.80 ± 0.81(595)	49.98	26	0.003
Weekdays vs. holidays					
Difference	0.22 ± 0.43(176)	0.36 ± 0.54(580)	32.83	25	0.135
Wake-up time					
Weekdays	6.85 ± 0.76(181)	7.02 ± 0.52(600)	46.35	22	0.002
Holidays	7.26 ± 0.76(181)	7.60 ± 0.81(594)	44.30	28	0.026
Weekdays vs. holidays					
Difference	0.41 ± 0.70(181)	0.58 ± 0.61(592)	36.08	34	0.372
Sleep duration					
Weekdays	9.65 ± 0.68(176)	9.58 ± 0.69(585)	27.15	37	0.882
Holidays	9.83 ± 0.64(180)	9.80 ± 0.78(587)	23.00	33	0.903
Weekdays vs. holidays					
Difference	0.19 ± 0.62(175)	0.22 ± 0.74(574)	33.49	45	0.897

On days when children went to kindergarten, 10 minutes was the most common amount of time during that they were exposed to sunlight from time they had breakfast (or woke up) until the time when they arrived at kindergarten, with 47.7% of 2-year-olds and 41.3% of 3 to 5-year-olds giving that response.

### Frequency of having breakfast

A total of 96.2% of 2-year-olds and 95.2% of children aged 3 to 5yearsate breakfast regularly at set times every day or nearly every day (95.5% for all infants aged 2 to 5years, Table 1). With respect to the number of times per week when children had a nutritionally well-balanced breakfast consisting of a staple food (carbohydrate), a main dish (protein), and a side dish (vitamins and minerals), 21.4% of 2-year-olds and 26.6% of 3-5-year-olds (25.1% for 2 to 5years, Table 1) had a balanced breakfast every day, whereas 20.3% of 2-year-olds and 21.3% of 3-5-year-olds (21.3% for 2 to 5years) had a balanced breakfast 4 to 5days/week, and 30.2% of 2-year-olds and 29.5% of 3-5-year-olds (30.0% for 2 to 5years) had a balanced breakfast 0 to1days/week.

The mean index of Trp taken at breakfast after the correction was 113.3 ± 41.69 (mg) for 2-year-olds and 119.2 ± 39.16 (mg) for 3-5-year-olds, while the mean amount index of Vi-B6 taken by 2-year-olds and 3-5-year-olds was 0.17 ± 0.10 (mg) and 0.18 ± 0.10 (mg), respectively.

### Circadian typology (M-E score) and several factors

**Figure 3:**
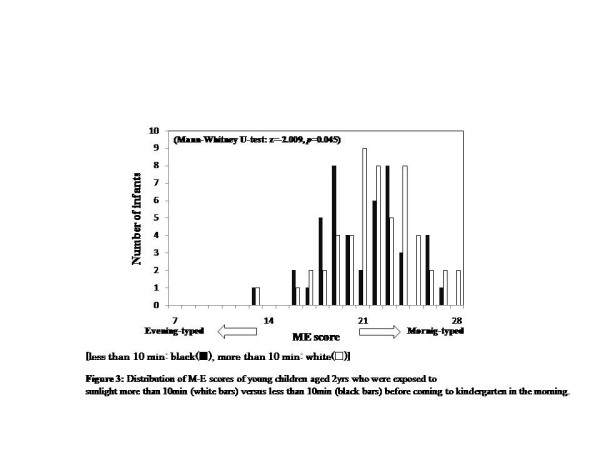
Distribution of M-E scores of young children aged 2 years who were exposed to sunlight for more than 10min (white bars) vs. less than 10min (black bars) before coming to kindergarten in the morning.

**Figure 4:**
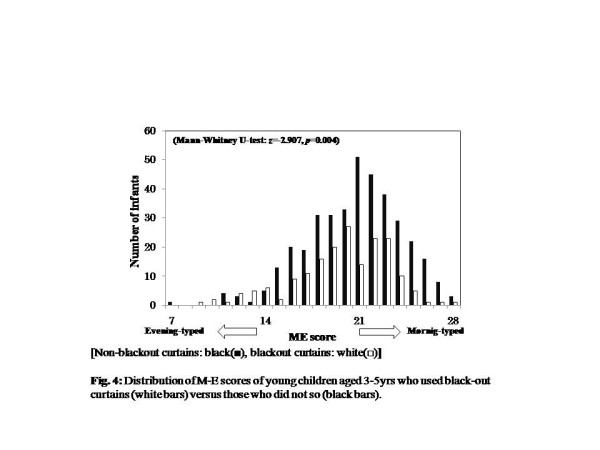
Distribution of M-E scores of young children aged 3 to 5 years who used blackout curtains (white bars) vs. those who did not use blackout curtains (black bars).

Looking at the relationship between the amount of time exposed to sunlight from breakfast time until reaching kindergarten, the M-E score was higher (more morning-typed) for 2-year-olds with more than 10 min exposure than 0 to 10 minutes exposure (Mann-Whitney U-test: 2years old, z = -2.009, *P* = 0.045, 0 to 10min = 21.67 ± 2.79, 10min = 22.51 ± 2.93, 3 to 5years old, z = -0.130, *P* = 0.897, 0 to 10min = 20.3 ± 33.53, 10min = 20.38 ± 3.51) (Figure 3). Children aged 3 to 5years who used blackout curtains in their bedrooms were more likely to be evening-typed than children who used non-blackout ones (Mann-Whitney U-test, for 2years, *z* = -1.264, *P* = 0.206, using non-blackout curtains, 22.23 ± 2.90, blackout curtains, 21.84 ± 2.85; 3 to 5years, *z* = -2.907, *P* = 0.004, non-blackout curtains, 20.68 ± 3.46, blackout curtains, 19.68 ± 3.51) (Figure 4).

**Figure 5:**
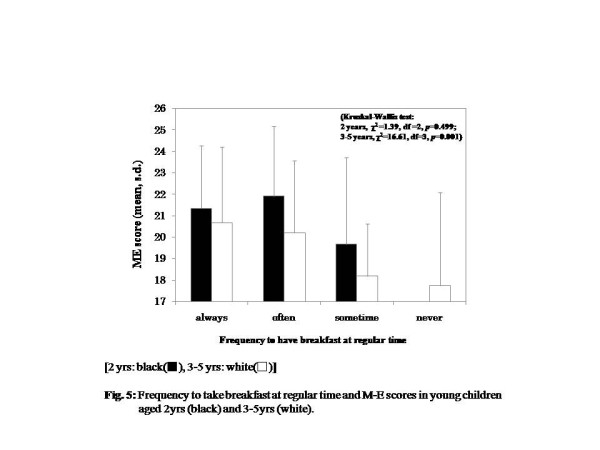
Frequency of having breakfast at a regular time and M-E scores in young children aged 2years (black) and 3 to 5 years (white).

**Figure 6:**
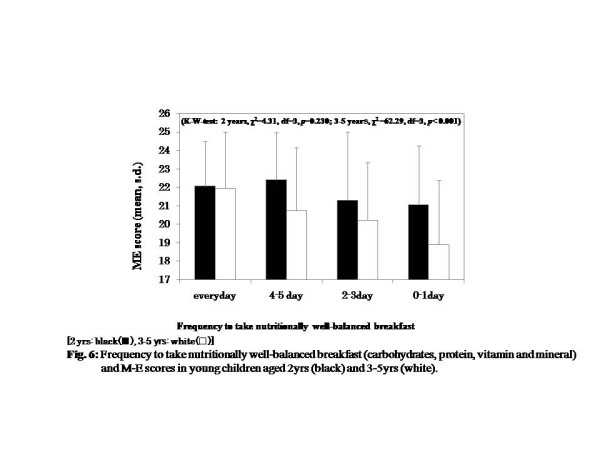
Frequency of having a nutritionally well-balanced breakfast (carbohydrates, protein, vitamin and mineral) and M-E scores in young children aged 2 years (black) and 3 to 5 years (white).

For children aged 3 to 5years, those who had breakfast at regular time almost every day and had breakfast that consisted of the three nutritional components were more likely to be morning-typed, but this trend was not seen in the 2-year-old age group (Figures 5 and 6) (Kruskal-Wallis test, effect of breakfast regularity, 2years, χ^2^ =1.39, df =2, *P* = 0.499; 3 to 5years, χ^2^ = 16.61, df = 3, *P* = 0.001, effect of nutritional richness, 2years, χ^2^ = 4.31, df = 3, *P* = 0.230; 3 to 5years, χ^2^ =62.29, df = 3, *P* < 0.001).

Three groups were made due to the amount of Trp and Vi-B6 intake: less than -1SD from mean, between -1SD to +1SD, and more than +1SD, for the analysis on the relationship between Trp amount of intake and M-E scores. Children aged 3 to 5years with greater Trp intake tended to be more morning-typed, although this trend was not seen in the 2-year-old group (Kruskal-Wallis test, 2years, χ^2^ = 1.68, df =2, *P* = 0.431; 3 to 5years, χ^2^ = 39.73, df = 2, *P* < 0.001). A similar relationship was also seen for Vi-B6 intake (Kruskal-Wallis test, 2years, χ^2^ = 1.63, df = 2, *P* = 0.422; 3 to 5years, χ^2^ = 33.38, df = 2, *P* < 0.001). Looking at the correlation coefficient between M-E score and Trp and Vi-B6 intake tended to give a slight but significant correlation of the r value of 0.25 (Trp intake: Spearman’s correlation coefficient = 0.25, *P* < 0.001, Vi-B6 intake: Spearman’s correlation coefficient = 0.25, *P* < 0.001).

### The integrated effect of Try and Vi-B6 intake and following sunlight exposure on M-E scores

Trp intake and Vi-B6 intake showed the similar relationship with M-E score. Such integrated analysis was done only for children aged 3 to 5years, because the number of data of those aged 2years or younger is too small to analyze.

**Figure 7:**
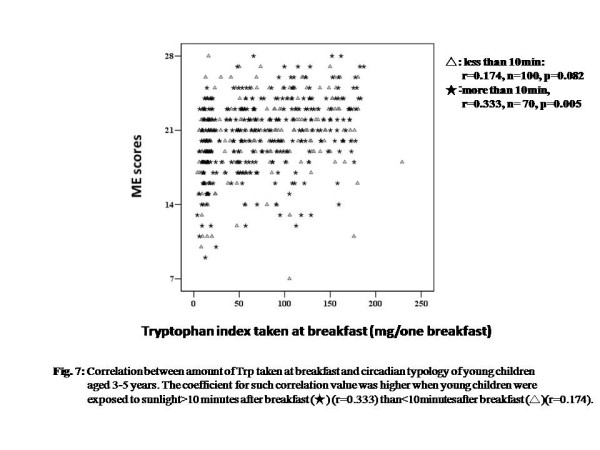
**Correlation between amount of Trp taken at breakfast and circadian typology of young children aged 3 to 5 years.** The coefficient for such correlation value was higher when young children were exposed to sunlight for more than 10 min after breakfast (★) (r = 0.333) than less than 10min after breakfast (△)( r = 0.174).

Figure 7 shows the relationship among exposure to sunlight on the way to nursery school or kindergarten, Trp intake, and M-E score. The amount of Trp intake positively and significantly correlated with M-E scores only for children who were exposed to sunlight for longer than 10min (Spearman’s correlation test, sunlight exposure more than 10min, r = 0.333, *n* = 70, *P* = 0.005; less than 10min, r = 0.174, *n* = 100, *P* = 0.082) (Figure 7). Dealing with both curtain use and sunlight exposure, the positive correlation tended to be stronger for children who used non-blackout curtain and children who were exposed to sunlight for longer than 10min and also used non-blackout curtains, than that for children who used blackout curtains and were exposed for less than 10min after breakfast (correlation value, sun exposure more than 10min before coming to nursery school: plus non-blackout curtain use, Spearman’s correlation test, r = 0.442, *P* < 0.001, *n* = 203,plus blackout curtain use, r = 0.172, *P* = 0.087, *n* = 100; sun exposure less than 10min: plus non-blackout curtain use, r = 0.287, *P* < 0.001, *n* = 147, plus blackout curtain use, r = 0.235, *P* = 0.050, *n* = 70) (Figure 7).

**Figure 8:**
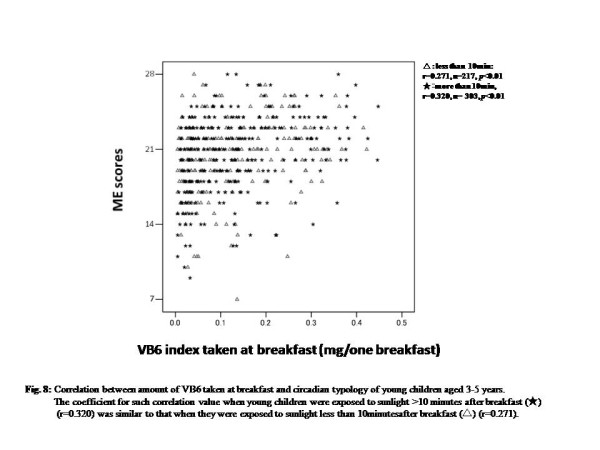
**Correlation between the amount of Vi-B6 taken at breakfast and circadian typology of young children aged 3 to 5 years.** The coefficient for such correlation value when young children were exposed to sunlight for more than 10 min after breakfast (★) (r = 0.320) was similar to that when they were exposed to sunlight for less than 10 min after breakfast (△) (r = 0.271).

Figure 8 shows the relationship among exposure to sunlight on the way to nursery school or kindergarten, Vi-B6 intake, and M-E score. The amount of Vi-B6 intake positively and significantly correlated with M-E scores irrespective of sunlight exposure (Spearman’s correlation test, sunlight exposure more than 10min, r = 0.320, *n* = 303, *P* < 0.001; less than 10min, r = 0.271, *n* = 217, *P* < 0.001) (Figure 8). Dealing with both curtain use and sunlight exposure, the positive correlation tended to be stronger for children who used non-blackout curtains and were exposed to sunlight for longer than 10min and also used non-blackout curtains than that for children who used blackout curtains and were exposed for less than 10min after breakfast (correlation value, sun exposure more than 10min before coming to nursery school: plus non-blackout curtain use, Spearman’s correlation test, r = 0.396, *n* = 203, *P* < 0.001, plus blackout curtain use, r = 0.176, *P* = 0.079, *n* = 100; sun exposure less than 10min: plus non-blackout curtain use, r = 0.265, *P* = 0.001, *n* = 147, plus blackout curtain use, r = 0.290, *P* = 0.015, *n* = 70) (Figure 8).

**Figure 9:**
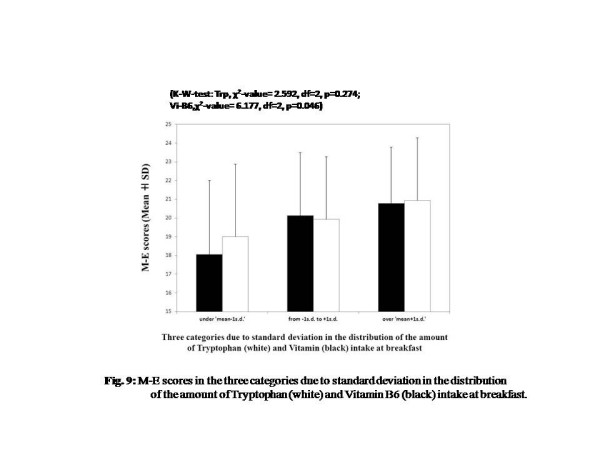
M-E scores in the three categories due to standard deviation in the distribution of the amount of Tryptophan (white) and Vitamin B6 (black) intake at breakfast.

In the comparative analysis among three groups due to standard deviation, a significant difference in M-E scores was observed not among the three groups for Trp intake (χ^2^value = 2.592, df = 2, *P* = 0.274), but among the three groups for Vi-B6 intake (χ^2^value = 6.177, df = 2, *P* = 0.046) (Figure 9).

## Discussion

Compared to 2-year-olds, 3 to 5-year-olds had lower average M-E scores and were more evening-typed, showing that there is a possibility that children can become more evening-typed as they get older, even starting from such an early age. With regards to breakfast habits, children aged 3 to 5 years tended to be more morning-typed if they ate a nutritionally-balanced breakfast made up of a staple food, main dish, and side dish every day. In other words, evening-typed children were likely to lack good sleep and did not eat nutritionally-balanced breakfasts. This trend was seen in children aged 3 to 5yearsbut not in children aged 2years. This may be a result of breakfast content becoming more fixed and children beginning to exhibit (and show large variation in) personal habits from 3years of age.

Furthermore, children showed a tendency to be more morning-typed if they ate breakfast with a high estimated Trp content. This study also confirmed a similar trend with estimated Vi-B6 content. Among essential amino acids, the Trp content in food is quite small, and thus it is necessary to make a special effort to consume a sufficient amount in one’s diet. Relatively large amounts of Trp are found in many ordinary foods such as dried bonito and other fish, fermented soybeans (*natto*), fish, meat, and eggs [15].

On the other hand, Vi-B6 is a coenzyme that is essential for the synthesis of serotonin and large amounts can be found in beef, pork or chicken liver, lean fish, and nuts and seeds [15]. Among fruits, bananas have a relatively moderate amount. As Trp and Vi-B6 are often contained in the same foods, children whose diet contains large amounts of Trp are also likely to receive larger amounts of Vi-B6 at the same time.

The amount of exposure to sunlight on the way to kindergarten and the type of bedroom curtains used also played a role in this process. Vi-B6 also acts as a coenzyme necessary for the metabolism of Trp into serotonin, and the synthesis of serotonin will be reduced without sufficient exposure to sunlight [8]. The results of this study supported these claims. Harada *et al.*[19] reported that college students who changed their bedroom curtains to blackout curtains in the summer either showed progressively later sleep onset times at night or, for those whose sleep phase was already delayed, their sleep-wake cycle became more irregular. For Japanese adolescents attending junior high school, the exposure to fluorescent lamps at home (including a high amount of blue lights with short wave of <480nm) during the night reduced the salivary melatonin level to one third of its potential level (under a light bulb) in a field experiment [20]. For such young children who are considered to be more sensitive to light than college and junior high students, the light environment including the type of bedroom curtains may be thought to strictly influence their sleep-wake cycles.

From these results, it might be suggested that a higher Trp and Vi-B6 intake may promote the synthesis of serotonin via light stimulation in the morning and have a natural sleep-inducing effect when converted to melatonin at night. As a result, it may help prevent a phase delay in young children’s circadian clocks and promote their morningness against the effects of the 24-h commercialization of society. In previous research using questionnaires, a link between Trp intake and M-E score was shown only for children aged 0 to 8years [6]. High levels of melatonin in the blood plasma at night are thought to have a large effect on sleep onset and the quality of sleep itself. Night-time blood plasma melatonin levels are many times higher in early childhood than during adolescence [21]. In the early years when children are learning and acquiring basic lifestyle habits, regular consumption of breakfast that contains sufficient amounts of Trp and Vi-B6 and proper ensuing exposure to sunlight might be necessary to help promote melatonin secretion at night, maintain sleep and mental health (related to serotonin as an anti-depressant agent), and the proper functioning of the circadian clock.

## Competing interest

The author(s) declare that they have no competing interests.

## Authors’ contributions

MY made Tryptophan Index 2009 and VitaminB6 Index 2009), analyzed data, and drafted this manuscript. OA collected questionnaire data, analyzed data and participated in discussion on the results. KW analyzed data and participated in discussion on the results. MK participated in discussion on the results. TN participated in discussion on the results. NT collected the data and participated in discussion on the results. HT made Tryptophan Index 2009 and Vitamin B6 Index 2009, analyzed data. TH supervised this study and drafted this manuscript. All authors read and approved the final manuscript.
